# Design and Implementation of a Prototype with a Standardized Interface for Transducers in Ambient Assisted Living

**DOI:** 10.3390/s150202999

**Published:** 2015-01-29

**Authors:** Enrique Dorronzoro, Isabel Gómez, Ana Verónica Medina, José Antonio Gómez

**Affiliations:** Department of Electronic Technology, Universidad de Sevilla, Avda, de Reina Mercedes S/N, 41012 Seville, Spain; E-Mails: igomez@us.es (I.G.); vmedina@us.es (A.V.M.); jgomezdte@us.es (J.A.G.)

**Keywords:** AAL, IEEE 21451, common standard interface, sensors, serious games

## Abstract

Solutions in the field of Ambient Assisted Living (AAL) do not generally use standards to implement a communication interface between sensors and actuators. This makes these applications isolated solutions because it is so difficult to integrate them into new or existing systems. The objective of this research was to design and implement a prototype with a standardized interface for sensors and actuators to facilitate the integration of different solutions in the field of AAL. Our work is based on the roadmap defined by AALIANCE, using motes with TinyOS telosb, 6LoWPAN, sensors, and the IEEE 21451 standard protocol. This prototype allows one to upgrade sensors to a smart status for easy integration with new applications and already existing ones. The prototype has been evaluated for autonomy and performance. As a use case, the prototype has been tested in a serious game previously designed for people with mobility problems, and its advantages and disadvantages have been analysed.

## 1. Introduction

Monitoring systems have traditionally been wired systems. Wireless technologies are changing this tendency, as shown in many scientific papers [1,2]. They are becoming popular in Ambient Assisted Living (AAL). Wireless Sensor Networks (WSN) are becoming increasingly important for monitoring patients both in the clinical setting and at home. WSN are fundamental in AAL since these smart systems, tailored to users’ needs, collect information about users and their ambience in order to provide personalized feedback.

WSNs consist of spatially distributed, autonomous sensors (sensing nodes) that cooperate to monitor physical or environmental conditions, such as temperature, sound, vibration, pressure, *etc.* They have several advantages over traditional wired systems: ease of use, reduced risk of failure, reduced user discomfort, enhanced mobility and lower cost of care delivery. As previously stated, the use of standards is not common in WSNs, thereby reducing the interoperability of the actual solutions. Using a standard is especially important in AAL applications where there are many different kinds of sensors.

The aim of the research presented in this paper has been to design and implement a prototype for transducers with a standardized interface that can be applied in the AAL domain. To this end, the next section, Section 2, provides a brief introduction to AAL systems, presenting the European AAL roadmap and typical AAL system implementations. Section 3 describes the standards used for these types of systems, while Section 4 describes in greater detail Standard IEEE 21451, the one used for the design of the prototype presented in Section 5 of this paper. Section 6 includes the evaluation of the prototype and the integration in a use case in Section 7. The paper concludes with the results and discussion.

## 2. AAL Systems

AAL, as defined in [3], refers to intelligent systems of assistance and represents a paradigm shift—in society as well as technology—that will walk hand in hand with “human-centred computing”, with the emphasis laying on user friendliness, situation awareness and distributed service support for human interaction. Their emergence provides a solution for the aging population. From this perspective, different projects have addressed the economic and social problems raised by aging populations. The most popular solutions for addressing the problem are those based on monitoring people at home, which can reduce the costs of caregivers, trips to clinics or hospitals, *etc.* It also makes them more independent, increasing their quality of life.

### 2.1. Components of AAL Systems

The AAL system structure is defined in [4], which presents a roadmap for AAL. It describes the main trends and analyses them from a demographic, economic and technological point of view. This document defines a reference architecture model that has been presented in the European project MyHeart [5] for remote patient monitoring.

**Figure 1.**
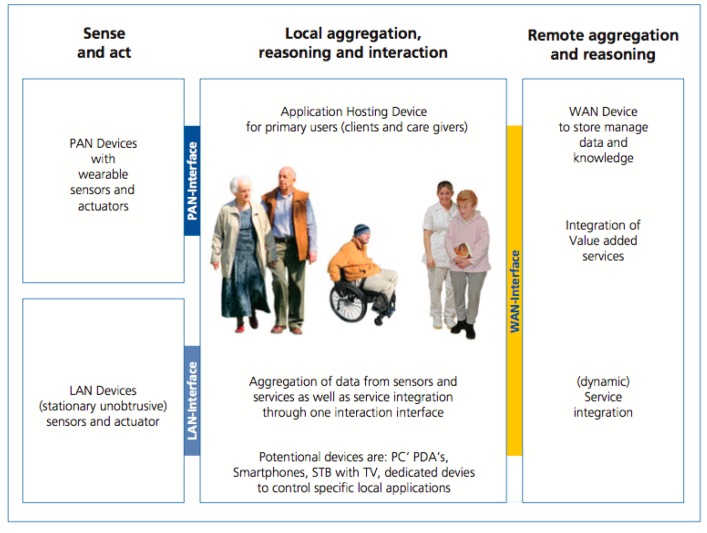
Structure of an AAL system as defined by the AALIANCE Ambient Assisted Living Roadmap (Ambient Intelligence and Smart Environments) [4].

One of the contributions of this document is the scheme presented in Figure 1, a three-layer networking approach to enable communication and connectivity between devices and services in the area of AAL. It is divided in the following components:

- Personal Area Network (PAN) and Local Area Network (LAN) devices: sensors and actuators that can be worn by the subject and stationary sensors located in the environment.
- PAN and LAN interface: for the connectivity, communication and data exchange between PAN and LAN devices with the Application Hosting Device.
- Application Hosting Device: in charge of four main tasks, communication with the PAN and LAN devices, communication with Wide Area Network (WAN) services, data storage and interaction with the user.
- WAN Interface: connects the Applications Hosting Device with external services via Internet.
- WAN Services: services that process, store, and execute actions with the obtained data.

### 2.2. Solutions in the AAL field for Monitoring of the Elderly

As previously stated (Section 2) AAL systems are presented to solve the problems arising from the increasing aging population. In this area there are two main solutions for monitoring the elderly at home: wearable sensors and smart homes. Both solutions are based on the use of sensors to gather data from the subject and/or the environment. The type of sensor, its interface, the transmission technology, *etc.* varies for each situation, making integration a challenge.

#### 2.2.1. Wearable Sensors

Although a wearable sensor cannot be an AAL solution on its own, it can be part of one by providing relevant data about the subject. This type of solution involves one or more sensors that are always carried by the subject. Nowadays most of the population uses a smartphone, which integrates different kinds of sensors such as accelerometers, gyroscopes, global positioning systems (GPS), *etc*, but there are also sensors that can be in the normal clothes that we wear.

Wearable sensors are used for many purposes [6]:

- Assisted living, activity recognition, the use of wearable sensors for activity recognition is usually based on the use of one or more accelerometers [7], for fall monitoring [8] and other kinds of applications.
- Continuous health and behavioural monitoring, where Wearable Health-Monitoring Systems (WHMS) allow the monitoring of physiological measures [9].
- Rehabilitation, wearable sensors, such as accelerometers [10] are used to provide support in rehabilitation processes.

Wearable sensors can provide several measures, for example, blood glucose, cardiac activity, blood pressure, *etc.* They go wherever the user goes; this means that the user will not go out of the area covered by the sensors. They don’t require expensive installation or maintenance. On the other hand, they require the user to be a very active part of the system. The user has to remember to carry the sensors every day and, as they are powered by batteries, their autonomy is low, and they must be recharged quite often.

#### 2.2.2. Smart Homes

The three levels of solutions for monitoring people at home are described in [11]. Smart houses are on the highest level. Health Houses, equipped with a set of sensors are able to evaluate many different variables. There are several home monitoring implementations [12,13] using a high level solution:

- The multipurpose in-home monitoring platform presented in [14] implements a monitoring system based on ZigBee. ZigBee and Bluetooth are the most popular transmission technologies used for AAL and health applications [1] due to their low power consumption. A “sensor common interface” has been developed for this platform. The main function of this interface is to separate the radio technology from the measurement hardware. It has been noted that the lack of common standardised communication interfaces is still a huge problem for WSN developers. Other issues such as acceptability problems have also been encountered. For instance, bed sensors were excluded in the first trial because of the anxiety caused by the technology.
- Another solution [15], also based on ZigBee wireless sensors, introduces two wellness functions to determine the wellness of the elderly. It is difficult to define the term wellness completely because the term changes according to different influential factors such as culture, experience, belief, religion, context, *etc.* They estimate the wellness of a person based on detecting abnormalities in the normal duration of the tasks.
- Another approach to home monitoring uses the electrical power line to perform the activity recognition [16]. Based on the idea that daily activities are strongly associated with rooms and electrical activities detected in them. It is important to assign weights to each electrical device based on the context. For instance, turning on the electric hob is more indicative of the activity of cooking dinner than turning on the kitchen light.

#### 2.2.3. Integration

The solutions in the field of AAL involve a heterogeneous set of devices. The integration of these devices is an important problem to be addressed. The interoperability of the systems facilitates the integration of the components by making possible the information exchange.

For the house environment, knowing the importance of the interoperability, the Ambient Assisted Living Joint Programme supported the action called “Support Action Aimed at Promoting Standards and Interoperability in the Field of AAL” [17]. Several projects (openHAB [18], universalAAL [19]) and initiatives (Home Electronic System [20], Open Service Gateway Initiative (OSGi) [21]) have focused in this area. They provide support to a set of devices from different manufacturers. However, there is still the needed of supporting a wide range of sensors, specially support for OEM sensors. In the healthcare field, there are also relevant organizations working towards the interoperability of mHeatlh products, such as Continua Alliance [22].

As shown there are many projects and initiatives that work on the standardization of different levels and fields, but some publications emphasize a lack of standardized interfaces for heterogeneous sensor networks, for AAL or other applications, as stated at [11,14,23,24]. The lack of standardization of the sensor interfaces decreases the interoperability as it increases the efforts of data exchange due the need of implementing proprietary interfaces. The prototype presented in this paper proposes a solution to promote traditional sensors, with a non-standardized interface to a smart status, by providing a standardized interface and access to a user network.

## 3. Standards for WSN in AAL Systems

The main point of the use of standards is to provide a standardized interface for sensors and actuators to encourage the integration of several AAL solutions by making easy the interoperability among them. In the literature, one finds two main standards that provide this interoperability for WSNs: IEEE 11073 defined for medical devices and, the one used in this paper, IEEE 21451 defined for transducer networks and which is not limited to the medical field.

### 3.1. IEEE 11073

The ISO/IEEE 11073 standards enable communication between medical, health care and wellness devices and with external computer systems. They provide automatic and detailed electronic data capture of client-related and vital signs information, and device operational data. This standard has been used by some authors [25,26] and was dismissed at [27] because it is too burdensome for systems with low memory requirements and low consumption and requires high amounts of available RAM, large packet length and it does not provide plug-and-play support. This standard was updated in 2010 with a new profile added to deal with these specific needs. ISO/IEEE 11073—part 20601 [28] resolves all the previously mentioned issues.

### 3.2. IEEE 21451

The IEEE 21451 [29] standard has been designed to standardize the definition of Transducer Electronic Data Sheets (TEDS) for each transducer and the data sending and reception and transducers description in instrumentation systems and control/field networks such as WSNs; it is not restricted to a single transmission technology and can operate with the most popular ones (ZigBee, Bluetooth, *etc.*).

This standard covers the standardization needs in Body Area Networks (BANs). It defines a standard interface for sensors and actuators providing flexibility and plug-and-play support when connecting different types of transducers within a network. The eight sections in which the standard is divided give us an idea of capabilities and the wide range of technologies covered in its definition:

- 21451.0, defines the basic functions required to control and manage smart transducers, common communications protocols, and media-independent TEDS formats.
- 21451.1, defines a network-neutral application model that will reduce the effort in interfacing smart sensors and actuators to a network.
- 21451.2, defines a digital interface for connecting transducers to microprocessors.
- 21451.3, defines a digital interface for connecting multiple physically separated transducers that allows multidrop, hot swapping, self-identification and configuration.
- 21451.4, defines the protocol and interface that allows analogue transducers to communicate digital information with an IEEE 21451 object.
- 21451.5, defines a standard for wireless communication methods and data format for transducers.
- 21451.7, defines data formats to facilitate communications between radio frequency identification (RFID) systems and smart RFID tags with integral transducers.

The wide range of available technologies and functionality makes this standard suitable for many different situations. As it is more general than IEEE 11073, in this work, IEEE 21451 has been used for the design and implementation of the prototype, which is presented in the following sections.

## 4. Basic Structure of the IEEE 21451 Standard

**Figure 2.**
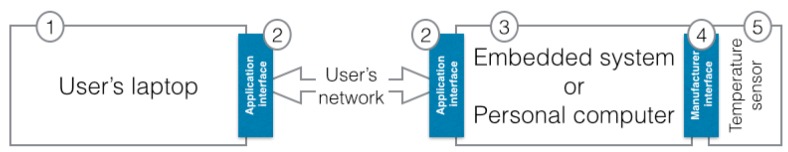
Gathering the data from the temperature sensor (5) requires the programming of an embedded system or personal computer (3), an application at the user's laptop (1) and two interfaces, one (2) for the communication between the user's laptop and the embedded system and other to access the sensor (4).

Let's think about the implementation of a system that triggers an alarm on your laptop when the temperature provided by an external sensor goes over a set threshold. In a traditional system the sensor must be connected to another computer or embedded system (points 1, 2 and 3 of the Figure 2). The sensor interface is proprietary of the manufacturer and it is different for each manufacturer. This means that the embedded system or personal computer must implement this specific interface (points 3, 4 and 5 of the Figure 2). This intermediate system must also implement the methods for polling data from the sensor and sends it to the user's laptop. Again we require a new interface for this communication. The data has to be sent to the user's laptop, but to perform this action the commands to access the data and its structure must be specified. Finally, a user's application is required to read the data from the network and trigger the alarm. The aim of the use of 21451 is to standardize the whole process making the integration between sensors and applications much easier.

As shown earlier (Section 3), IEEE 21451 is used more widely than the IEEE 11073 standard. This standard aims to promote transducers to a smart status by defining a smart transducer interface. For the implementation of the prototype we have considered just the implementation of sensors, not actuators, that is why from now on all the descriptions will refer to sensors, even the standard contemplates also the interface for transducers (sensors and actuators). The standard considers that a sensor is smart when it has the following three features:

- It is described by TEDS. TEDS are fundamental in the standard as they define the sensor behaviour.
- Data and control are digital.
- Triggering, status and control are provided, this means that it must be possible to control them when reading data, checking the current status and adjusting the settings of the sensor.

**Figure 3.**
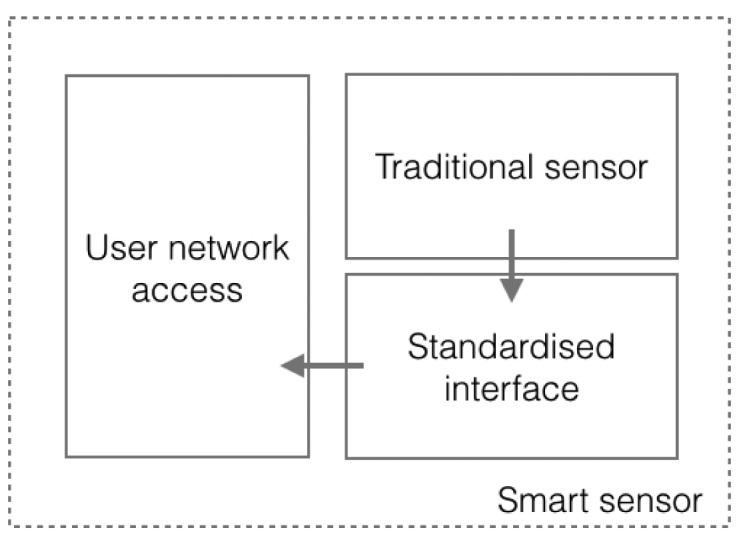
Smart sensor structure, as defined by IEEE 21451.0. The structure consists of three elements: a traditional sensor, a standardized interface and user network access to provide network connectivity.

To achieve this goal the standard defines two modules, which provide a standardized interface and network capabilities for the sensor (Figure 3). These modules are the Transducer Interface Module (TIM) and the Network-Capable Application Processor (NCAP).

Making an analogy with the temperature sensor example, the smart sensor covers points 2, 3, and 5 in Figure 2. To trigger the alarm, using the standard, the implementation is reduced to the implementation of the user's application on the computer laptop, which has to implement the application interface (points 1, 2 in Figure 2).

### 4.1. Transducer Interface Module

This contains the interface, signal conditioning, data conversion and, sometimes, the transducer. The TIM provides the sensor with the capabilities to be integrated into a system without needing drivers. To achieve this requisite the TIM uses the TEDS and a command set. TEDS describe how to access the TIM and its behaviour. The command set allows the acquisition of the data and TEDS read and writing. For example, one service is to periodically send data to the NCAP and there is a specific command to establish this kind of operation.

**Figure 4.**
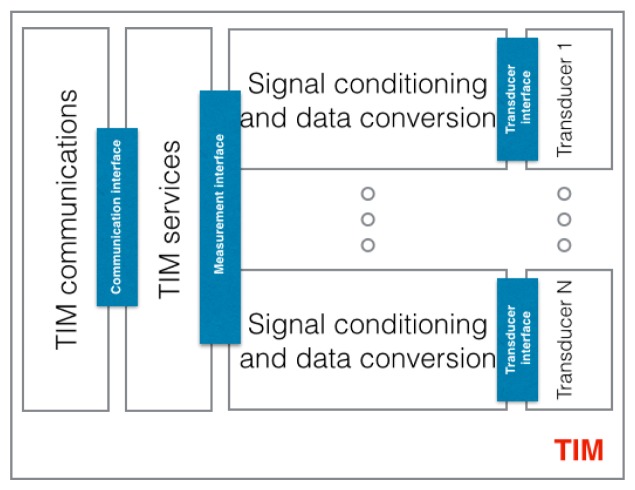
TIMs’ general functional structure. TIM communications provides connectivity between the TIM and the NCAP. TIM services implement services to the NCAP to control or get/send data to the transducers. Several transducers can be connected to one TIM.

Basically, all the TIMs can implement a group of services defined by the standards. For example, it is obvious that there has to be a way to access the data provided by the TIM. Every manufacturer defines a set of commands to access this data, but these commands are different between manufactures. This standard defines a service to access the data and also a set of commands to read data from the sensor, in other words, to access the service. A TIM's structure is presented in Figure 4 and it implements the following functions:

- Communications: this communication module refers to the communication between the NCAP and the TIM. Basically, it is an interface that receives the NCAP commands that request a specific service and returns a reply once the service is executed.
- Services: providing services to the NCAP such as polling, calibration, description, *etc.*
- Signal conditioning and data conversion: conditioning the signal provided by the sensor and conversion from analogue-to-digital and from digital-to-analogue.

The TIM is able to support more than one sensor, in this case a common interface for each sensor is provided. In the example used to illustrate the standard components when a command, coming from the NCAP, requesting data from the sensor arrives at the communication module, the corresponding service reads the data of the temperature sensor that is connected to the analogue-to-digital converter. The TIM solves points 5, 4 and part of point 3 in Figure 2. The TIMs implement the manufacturer's interface to acquire the data from the temperature sensor (point 4). This includes part of point 3 because it implements the standard services to acquire the data from the sensor, but it does not provide access to the user's network, so basically it acts as a bridge that connects the standardized services to access the data from the temperature sensor to the one provided by the manufacturer. Once the data is read it is sent back to the NCAP.

### 4.2. Network Capable Application Process

**Figure 5.**
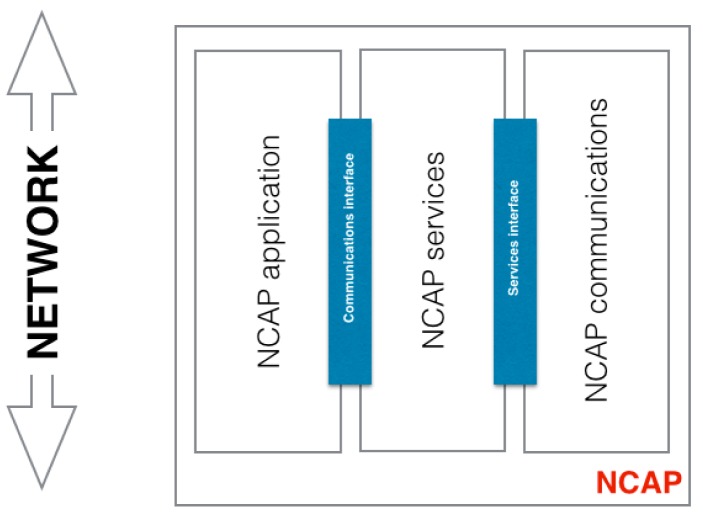
NCAPs’ general functional structure. NCAP communications provide connectivity between the TIM and the NCAP, the NCAP services are used to control and get/send data from/to the TIM and NCAP application provides services to the user.

The NCAP mediates between the TIMs and the user network. It provides a network interface providing the communications and services to communicate the sensors with services in the user network. The TIM by itself does not have the capability of being accessed by a user network, it just provides an interface to access the implemented services. The NCAP is in charge of this capability; its aim is to provide an access to the sensor through a user network. For example, if a user wants to read the temperature value provided by a sensor the NCAP can transmit that information using the WIFI network. Then an application can present the data in the user's computer. The NCAP structure is presented in Figure 5 and it implements the following functionality:

- Communications: this communication module also refers to the communication between the NCAP and the TIM. It is analogous to the communication module at the TIMs.
- Services: provides services to the NCAP application such polling, calibration, description, *etc.* Basically this module receives the application's requests and transmits the request to the TIMs. For instance, the NCAP has an application that requires reading the data from a contact sensor. The application requests the data to the NCAP services module and the service module requests the data from the TIMs.
- NCAP Application: provides services to the user network.

The behaviour of the NCAP is easier to understand using the example given at the beginning of this section. The NCAP application is defined to send data from the temperature sensor via the user network, this means that it is able to send data through the user's WIFI network, but first it needs to read the data from the temperature sensor. To do so it accesses the corresponding service (defined by IEEE 21451). The service requests the data to the sensor by sending the command to the TIM via the communication module. The same module that receives the reply and the service sends the data to the NCAP application. Back to Figure 2, it implements the part of point 3 that is not covered by the TIMs, in other words, the access to the user network (point 2 and part of point 3 of Figure 2).

### 4.3. Transducer Electronic Data Sheet

Finally it is important to reiterate that the standards use TEDS. TEDS are electronic data sheets that provide descriptions and access to a TIM and the transducers associated with a TIM. They store the transducer identification, data range, measurement units, calibration, correction data, manufacture-related information, *etc.* The design and implementation based on the previously presented AAL structure and the TIM and NCAP scheme are described in the following sections.

Let us go back to the example given at the beginning of this section. The different elements needed for the implementation of the system are covered by the structure of this standard. The process goes through the following steps (the detection and identification of the sensor is omitted as it is illustrated in detail in Section 7):

(1) The laptop application requests the data from the sensor. This means that the application communicates with the NCAP application module, via WIFI and using a standard command, to request data from the temperature sensor.
(2) The NCAP applications request the data from the NCAP service in charge of this specific function.
(3) The NCAP service requests the data from the pertinent TIM by sending the command defined by the standard through the communication module. This command specifies the TIM and the sensor in the TIM.
(4) The TIMs read the data from the sensor and the data goes all the way back.
(5) The application reads the data and triggers the alarm if it is over the threshold.

This process is transparent to the application that just requests a service from the NCAP. The application just needs to implement the application interface (point 2 of Figure 2) and the smart sensor will be in charge of requesting the data to send the requested temperature data. Any module of the system could be replaced by a new one and, as long as it is compliant with the standard, no change is needed in the application.

## 5. Standard Based Prototype Design

This section describes the design followed to implement the prototype. The design is split into two phases. The first phase describes the mapping of the IEEE 21451 standard with the structure of the AAL systems described in Section 2.1. The second phase presents the design of the two main components, TIM and NCAP (Section 4), of the standard IEEE 21451 and the physical components. Finally it describes the transmission technology used between the TIM and NCAP and an http server that provides access to the NCAP through the user network.

### 5.1. IEEE 21451 Mapping

Figure 1 presented the three-layer networking approach to enable communication. The connectivity between devices and services in the area of AAL and their components have already been described (Section 2.1). This structure has been used in order to design the prototype; the idea is to cover all the three layers with the standard IEEE 21452. To achieve this goal the following mapping has been done:

- Sense and act: This layer of the roadmap is implemented by the TIMs, as Personal Area Network (PAN) and Local Area Network (LAN) devices. IEEE 21451 upgrades a sensor providing it with plug-and-play features. Using this feature there is no need to differentiate between LAN and PAN sensors. *i.e.*, as soon as a person is close (inside the network area coverage) to an environmental sensor (LAN sensors) the sensor can be incorporated dynamically to the Application Hosting Device. This means that the subject does not need to do anything, just get close enough to the sensor and the Application Hosting Device will start to receive data from it. Sensors in this layer are mapped as TIMs in the standard.
- Local aggregation, reasoning and interaction: The Application Hosting Device is implemented by the Network Capable Application Process (NCAP). The data from the TIMs (sense and act layer) are aggregated at the NCAP. The NCAP should be capable of processing the data to perform the reasoning required by this layer.

To aggregate the data a set of services and the communication between layers has to be established. We consider the use the IEEE 21452.0 section to define the services and the IEEE 21451.5 section for the communication. The services provided to the remote aggregation and reasoning layer are covered by the same section of the IEEE 21451.0 standard. The communication is done via HTTP protocol.

- Remote aggregation and reasoning: Consisting of WAN Services that are implemented by the user application.

There are two interfaces that provide communication among the three layers:

- PAN and LAN interface, the services interface is provided by the TIM specification. The communication interface is defined in IEEE 21451.5.
- WAN Interface, this interface is an implementation of the Hypertext Transfer Protocol (HTTP) Application Programming Interface (API) defined in IEEE 21451.0.

### 5.2. Components Mapping

**Figure 6.**
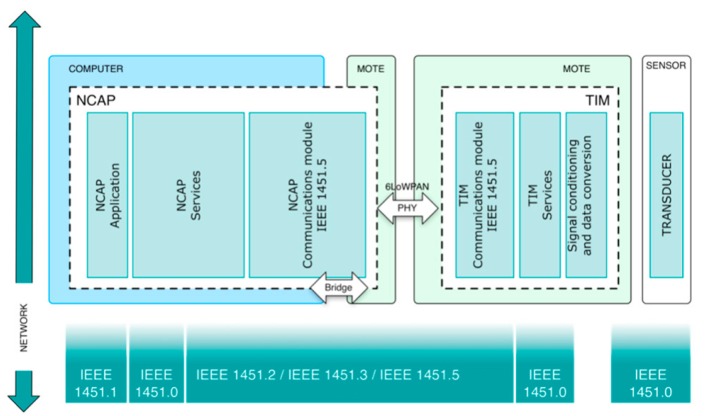
General structure of the IEEE 21451 prototype. It illustrates the mapping between the components that implement the prototype and the modules and sections defined by the standard. 5.2.1. Transducer Interface Module

This section describes the steps to design the prototype: the system structure, choosing the components and mapping the functionality of the standard. The prototype has been designed by taking into account, what has been previously described in Section 4 and following the structure defined by the IEEE 21451 standard (Figure 6).

As previously stated (Section 4.1) the TIM provides the sensor with a common interface based on the TEDS and a command set. The design and implementation of the TIM has been done using a CM5000 Advanticsys mote [30], which uses TinyOS as the operating system and is programmed using nesC and two sensors.

- Mote: the CM5000 mote is an IEEE 802.15.4-compliant wireless sensor node based on the original open-source TelosB/Tmote Sky platform. This mote uses TinyOS [31], which is a free and open source operating system; it is component-based and designed for wireless sensor networks.
- Sensors: two different sensors are included in the prototype, a temperature sensor and an accelerometer sensor. The temperature sensor is built in the mote and the accelerometer sensor is connected via the Analog-to-Digital Converter of the mote.

TinyOS [32], as previously mentioned, is an open source, flexible and application specific operating system. It includes a library of components for accessing the sensors, network communication protocols, *etc.* These components can be assembled based on the requirements of the specific application to be developed. TinyOS motes have been used in several research lines. There are many important companies involved in the development, Crossbow Technology Inc. since 1995, Advanticsys that operates worldwide, *etc.* The TinyOS community is growing, and with it, the support includes thousands of developers and users in dozens of countries, plus hundreds of companies, universities, and government institutions. Another important aspect of TinyOS is that it is more efficient in terms of power consumption than other popular operating systems, such as MOS and Contiki [33].

These components have been mapped with the standard IEEE 21451 as follows:

- Communication module. Two protocols are used for the implementation: IEEE 802.15.4, implemented by the RF chip of the mote, and 6LoWPAN implemented, in nesC code, at the flash memory in the mote.
- TIM services. They have been implemented, in nesC code, at the flash memory in the mote.
- Signal conditioning and data conversion. It is performed by the ADC integrated in the mote.
- Sensors:
  ○ Temperature sensor, this sensor is integrated in the mote. It is connected to an internal ADC. This means that the access to this sensor is done in the same way that an external sensor. The sensor model is a Sensirion @SHT11.
  ○ Accelerometer sensor, the accelerometer model is ADXL335 [34] from Analogue Devices. It is a small, thin, low power and three-axis accelerometer with signal conditioned voltage outputs. The accelerometer is composed of three sensors as it provides measurement for three axes (x, y, z). The sensor is connected to a different ADC using the EX1000 extension board.

#### 5.2.2. Network Capable Application Process

The NCAP mediates among the TIMs and the user network. The implementation of the NCAP has been done by using the CM5000 mote and a standard personal computer. The mote acts as a bridge for data reception and transmission while the rest of the tasks take place in the computer.

- Mote: The CM5000 is the same mote model as the one used in the TIM.
- Computer: A standard personal computer with a Linux distribution, Ubuntu 10.04 Long Term Support (LTS) and programmed in JAVA.

Its functionality is more complex than the one performed by a TIM. Using a computer as the NCAP reduces the mobility of the system but increases the available memory and processing capabilities, which improves the performance and complexity that the NCAP requires. Implementing this functionality in a mote is hard due to the memory and power limitations. The computer also provides a gateway to the user network. The NCAP has been mapped in a similar way to the way it was done with the TIM. The components’ functions for the NCAP are:

- Communication module. It is similar to the TIM design.
- NCAP services. These services have been implemented, using JAVA, at the computer.
- NCAP application. It depends on a specific implemented application. As this papers presents a general prototype that is not used for a specific scenario this application module is not covered.

#### 5.2.3. Transmission Technology

The 21451.5 section of the standard describes the different wireless transmission technologies that can be used to communicate the NCAP and the TIM. For our prototype, 6LoWAN was chosen. IP is used as it provides many advantages, as described in RFC 4919 [35]. The pervasive nature of IP networks allows the use of existing infrastructure, IP-based technologies already exist, are well-known, and proven to be working, tools for diagnostics, management, and commissioning of existing IP networks and IP-based devices can be connected readily to other IP-based networks, without the need for intermediate entities such as translation gateways or proxies.

In order to get all these advantages into WSN, with the challenge of reducing power consumption and the size of the devices, the Internet Engineering Task Force (IETF) group working on 6LoWPAN has emerged. The aim of this group is to develop a model that conforms IPv6 to the 6LoWPANs over the IEEE 802.15.4 standard. 6LoWPAN implementations have been used successfully in several WSNs [36,37].

There are some implementations of 6LoWPAN that use the specifications of the IETF working group. The three main implementations are analysed in [38]. Of these three implementations BLIP and Sicslowpan are the ones that have better performance and both are good choices for building the prototype. We have chosen BLIP [39] as it runs on TinyOS and has been implemented by the same developers of the IOS, University of California Berkeley.

#### 5.2.4. HTTP Server

The HTTP server implements the API defined by the standard. The aim of the API is to provide access to the NCAP to get the TEDS and data provided by the transducers. The data provided is formatted as XML in the prototype.

## 6. Evaluating the Prototype

This section describes the tests that have been done to analyse and study the behaviour and performance of the designed and implemented prototype. Three different types of tests have been made over the implementation:

- Compliance test, to analyze the correct behavior of the prototype.
- Performance test, to evaluate the efficiency of the prototype
- Autonomy test, to estimate the maximum autonomy of the system.

### 6.1. Compliance Tests

The aim of these tests is to analyse the implementation of the prototype by comparing the traffic generated with the theoretical one described in the documents that define the standard. That traffic has been used to determine the correct behaviour of the protocol based on the IEEE specification. The traffic has been captured by a protocol analyser (Wireshark [40]) and compared at byte level with the standard definitions.

**Figure 7.**
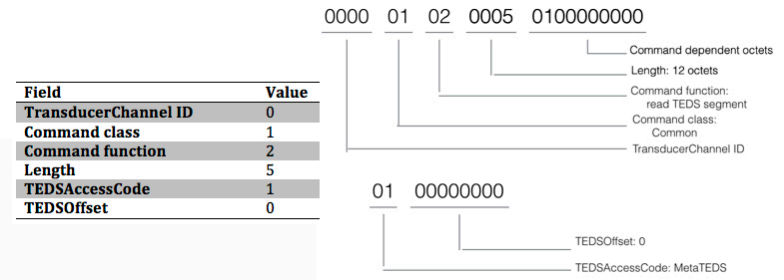
Compliance of the Read TEDS Segment command. The table provides the theoretical values defined in the IEEE standard. The data on the left presents and interprets the data captured during a real transmission.

For example, for the standard “command read TEDS segment” the data captured with Wireshark can be analysed and compared with the standard specification, Figure 7. On the right hand side of this figure the table shows the theoretical values for the fields that make up a typical “read TEDS segment” command as specified by the standard. The left part presents the array of bits captured by Wireshark grouped by the same fields that are included in the mentioned table. As one can see, these fields share the same values.

### 6.2. Autonomy Tests

It is important to determine the autonomy of the system, that is, how long the prototype can be acquiring and transmitting data without needing to recharge the batteries. The autonomy of the system is determined by the TIMs and these are powered with two AA batteries.

To carry out the tests a NCAP has been connected to a TIM. At the beginning of the test the NCAP requests to the TIM the corresponding TEDS. The tests were run three times for two configurations: without connecting a sensor, and connecting an accelerometer to the TIM.

After this initial process the NCAP requests the TIM to periodically send data. The data that is sent by the TIM is the data acquired for the battery sensor. As data arrives to the NCAP the time of arrival is recorded in epoch format. The epoch format measures the time in milliseconds since 1 January 1970.

Subsequently continuous data samplings are sent from the TIM at regular intervals. This data contains the battery status at the time of mailing. For each message received in the NCAP the battery status and the time of receipt of the message is logged. The test ends when the TIM receives no messages.

The results of this test are shown in Table 1. The number of hours between the initial time and the end time is provided in the following formula:

(1367987s−1367908s)/3600s=21.97h

The time difference between the start and end of the test is 21.97 h. The tests made with and without the accelerometer sensors provided the same results. The accelerometer had no impact on the total autonomy of the system. The autonomy is basically determined by the power consumption by the radio chip that is always active, as BLIP implementation keeps the mote at the awaken state.

### 6.3. Performance Tests

We conducted tests to study the performance of the implementation based on the number of nodes that make up the network and the sampling frequencies of each node. Because of the small number of motes, *i.e.*, TIMs available at the laboratory, a TOSSIM simulation was built to study the performance of the standardized prototype. The performance analysis was done using the following methodology:

(1) The protocol behavior was simulated and validated. TOSSIM does not implement BLIP, this is why the protocol behavior had to be simulated.
(2) Simulations of the communication between the NCAP and TIMS. These simulations were performed by TOSSIM using the validated simulation code of the first step. One simulation was conducted for the different values that the variables could take, for frequency: 30 ms, 60 ms and 90 ms and the numbers of TIMs were increased from 1 to 14. The top TIMs value was selected during the tests based on the low performance of the network when the number of TIMs was high. Instead of running many simulations with low message exchanges for each simulation, each TIM exchanged 2001 messages with the NCAP. The number of messages exchanged was set high to have a relevant number of transmissions to make the results given by TOSSIM simulator more accurate.
(3) Statistics of the number of packets lost in the transmission were gathered. The statistics results are based on the simulation made by TOSSIM.

**Figure 8.**
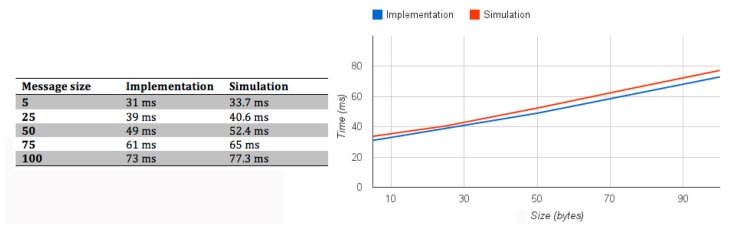
Protocol performance: implementation *vs.* simulation. The scenario presents two TIMS transmitting messages with different sizes. The table presents the delay due to the message composition and transmission for the implementation and simulation. The graph represents the same values to visualize them.

TOSSIM is a simulation tool, which can make simulations using the TinyOS code. The main advantage is that the same code that runs in the mote is used in the simulation, but there is a problem when using TOSSIM for evaluating the performance of the protocol. TOSSIM cannot simulate BLIP implementation. To make the performance study, BLIP traffic was analysed, and simulated in TOSSIM. The results of the simulation have been compared with the results of real tests (prototype test) to validate them. The table in Figure 8 presents the time (in ms) for the message exchange to get a TEDS by the NCAP. Different tests have been made by modifying the size of the messages. The time taken to complete these tests, in the simulation and the prototype, is presented in the table and graph of Figure 8. One can see that the results of the simulation and the prototype are similar.

**Figure 9.**
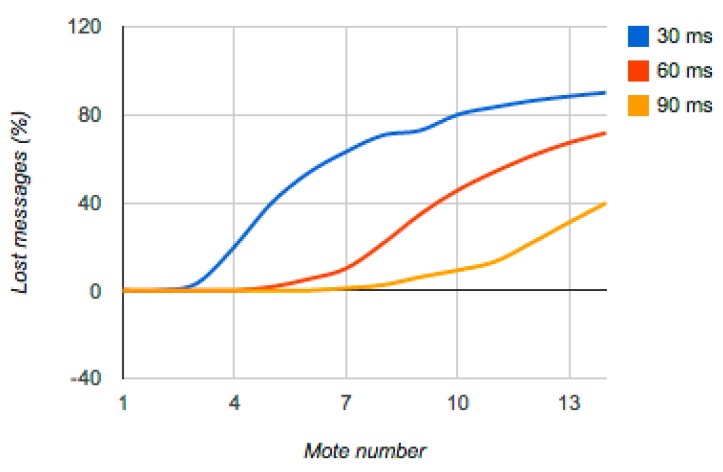
Lost messages due to the TIMs number. Data is provided for three sampling frequencies, 30, 60 and 90 ms. The percentage of messages lost due to collisions are showed on the Y axis while the number of TIMs is on the X axis.

Once we had tested that the BLIP simulation in TOSSIM behaved like the prototype, we conducted several tests in TOSSIM by modifying the number of motes (TIM) and sampling frequency. The results of these simulations are presented at Table 2 and Figure 9.

Figure 9 presents the number of messages lost, these per cent is calculated as an average of the 2001 messages exchanged by every TIM and the NCAP at the simulation. They provide the following information:

- For sampling values below 30 ms, the number of lost messages is greatly reduced when the network has no more than three TIMs. However for a higher number of TIMs the loss of messages is excessive, reaching up to 50% with only five TIMs.
- For sampling values of 60 ms for less than six TIMs the percentage of lost messages is reduced. Above seven messages the loss is quite high.
- For sampling values of 90 ms, message loss is low for a number of TIMs up to 10 TIMs. This sampling value provides 10 data values per second, which may be more than adequate for a large number of applications. As presented in [1] a typical application does not include more than three sensors. Increasing the number of TIMs over this value increased of additional loss by 10% for each new TIM.

## 7. Use Case Application

**Figure 10.**
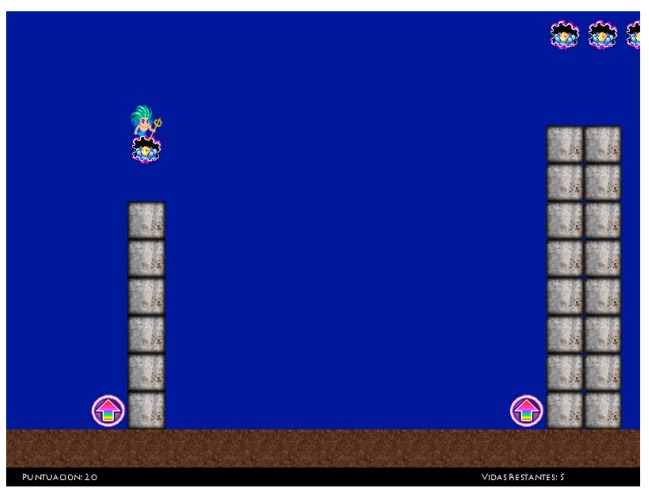
Aquaventure game screenshot. This screen shows a capture of the serious game Aquiaventure used as a use case.

The use case presents a study where a sensor compliant with the IEEE 21451 standard was integrated into a previously implemented system. This was done by using the designed prototype. The aim of the use case was to show the advantages and disadvantages of the use of the prototype. This use case is based in a serious game developed at the same Department of Electronic Technology of the Universidad de Sevilla, as a master project. Serious games are an emerging research area and they are used for several purposes such as rehabilitation [41], education and health [42], *etc.* The game used in this use case is a serious game called “Aquaventure” (Figure 10) designed for people with reduced mobility. The aim of the game is to stimulate eye-hand reflexes and increase awareness of the surroundings. This game was designed to be controlled with a keyboard or a push button to make the character jumps.

This section presents the integration of the serious game with the prototype. The main idea is to use an accelerometer as a controller for the game. The typical solution is to implement a driver that provides the interface between the game and the accelerometer. By using the prototype, the game can detect the new sensor and incorporate it in the game without any setup or configuration. The accelerometer only has to turn on.

### 7.1. Integration

**Figure 11.**
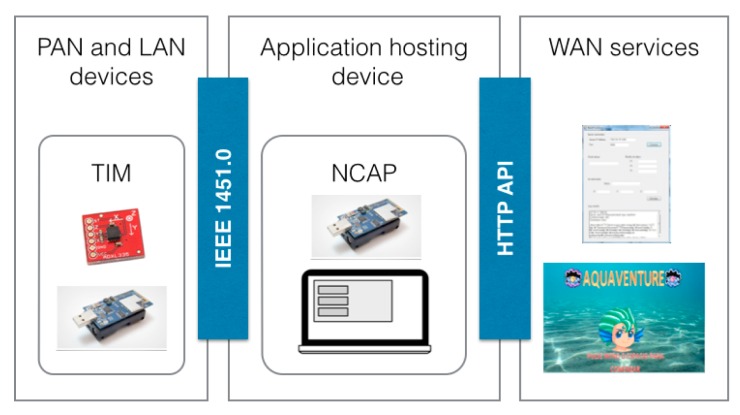
Aquaventure reference model based AAL reference model presented in Figure 1, including all the components described and defined in Section 5.

The system implementation and the integration with the game are done by following the system structure described in this paper (Figure 11). The reference model described in [29] is a reference for the design of the prototype. Based on this model the prototype implements the PAN and LAN devices and the application hosting device. The Aquaventure game and the NCAP interface application implement the WAN services.

The integration with the Aquaventure game has been done using an intermediate application (NCAP interface application), which connects this game with the WSN. This is done using the HTTP API defined by the 21451.0 standard. The NCAP interface application is in charge of communicating with the HTTP server integrated in the NCAP to discover new TIMs and transducers. The other purpose of the NCAP interface application is to acquire the data from the accelerometer and transform this reading into events to interact with the Aquaventure game.

### 7.2. Game Test

**Figure 12.**
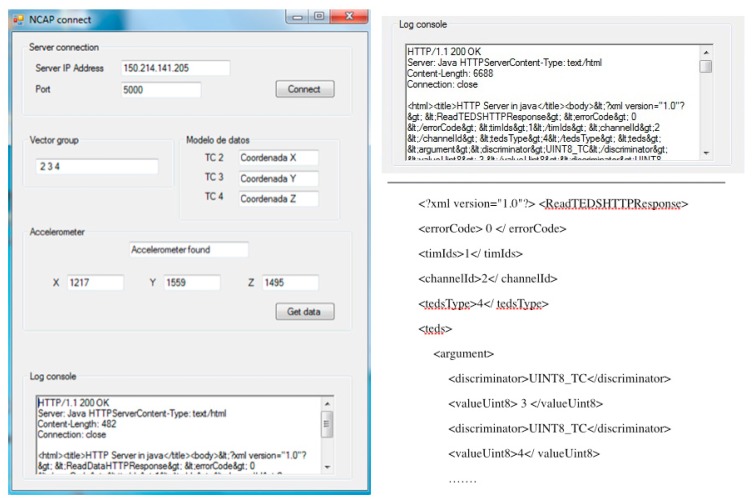
NCAP connect application. The application connects with the prototype (IP 150.214.141.205:5000), acquires the information and data from the sensors connected in the prototype and presents it in the front-end (vector group, data model, sensor data, *etc.*). The right side of the figure presents an example of the XML defined for a transducerChannel TEDS acquired by the NCAP connect application during a transmission.

The purpose of the test is to use an accelerometer as a controller of the game. By using the prototype the game can detect that an accelerometer is connected and start acquiring the data provided by the sensor. Based on this data a jump action can be triggered to interact with the game. The TIM and the accelerometer sensor are located in the user's hand. The NCAP, NCAP interface application (Figure 12) and Aquaventure game are running on a PC.

When the game starts the sensor is shut down, so there is no active controller. The NCAP application is running in the background and checks periodically for an accelerometer using the discovery process. Once the accelerometer is connected the discovery process performs all the steps. The accelerometer is detected, Figure 12 shows an example of the TransducerChannel TEDS acquisition log, and at this moment the acquisition of data begins. The data is acquired by the NCAP interface application and triggers an event when the x-axis measure exceeds a defined threshold. For this scenario the threshold has been set manually without any criteria as this is outside the field of the study. The events are sent to the game that performs a jump.

## 8. Conclusions

This paper presents a prototype that provides an open source interface which is easily adaptable to assisted environments. Given the advantages provided by WSNs: ease of use, reduced risk of failure, greater comfort, improved mobility, *etc.* the use of a standard provides a standardized interface that facilitates the integration of different kind of sensors and solutions.

The prototype presented has been designed using the IEEE 21451 standard, TinyOS motes and 6LoWPAN. IEEE 21451 provides a standardized interface; TinyOS motes is a free and open source operating system and hardware; and 6LoWPAN is a transmission protocol that includes the advantages of IP. This prototype includes two sensors, an accelerometer and a temperature sensor. An accelerometer is normally used as a body sensor, and a temperature sensor can be used as environmental sensor. This prototype can be used as part of the solutions for AAL systems by providing a standard interface and reducing the need for knowledge of proprietary interfaces to control and acquire data from sensors. As we mentioned in our discussion of the state of art (Section 2.2), some solutions [14] have had to implement their own common interfaces to access the sensors of the system.

The use of the prototype also contributes as a path towards the integration of the solution with other systems. There have been some studies to integrate IEEE 21451 with other standards, as HL7 and IEEE 11073 [43,44]. Three different types of tests have been made over the implementation:

- Compliance: The traffic generated by the implementation has been used to determine the correct behaviour of the protocol based on the IEEE specification.
- Autonomy: Power consumption tests have established the autonomy of the system at around 22 h. The autonomy is determined by the power consumption of the radio chip that is always active, as BLIP implementation keeps the mote in the awaken state.

The consumption of the radio chip implies that the sensors used are not an important factor due to the autonomy of the system. While experiments were also conducted with and without the accelerometer, the autonomy remains similar for all the tests, and the impact of the sensor is imperceptible. The 22 h of autonomy allow the subject to carry the sensors for one day without having to recharge the sensors that she or he is carrying. Static sensors deployed in the house or the environment could use an external power source.

- Performance: To study implementation performance, tests have been made based on the TIMs that make up the network and the sampling frequencies of each one. The system has been shown to be efficient in environments that have a high number of nodes (up to ten) and with high sampling frequencies (up to 90 ms). This is a high number of sensors (TIMs) for these systems. As shown in [1], the use of WSN applications usually involves two or three sensors.

It is a fact that the efficiency of the system and the number of TIMs for good performance is limited by the application. For example, if the data source is a temperature sensor located in a room, the impact of losing some data is not very serious as the temperature is unlikely to vary in a short period of time. In contrast, the data returned by a proximity sensor may vary in a short period of time, in that case the number of TIMs will have to be reduced to guarantee the minimum data loss to have a reliable system.

As TOSSIM cannot simulate BLIP, to do the performance study, BLIP traffic has been analysed and simulated in TOSSIM. The results of the simulation have been compared with the results of real tests to validate them. Finally, the prototype has been integrated in a serious game. Aquaventure is a game that was not developed to be used with the IEEE 21451 standard. As has been demonstrated, the integration with the prototype allows the game to detect when an accelerometer sensor enters the system and is able to acquire the data provided by this sensor to control the game. The game does not need to know the specific interface that the manufacturer has implemented in the sensor. Typically, without the use of the standard, the programmer has to use a specific driver and adjust the code of the application with the driver API to be able to acquire the data from the sensor. The proprietary interface is transparent from the implementation point of view. The developer just needs to know the interface defined by IEEE 21451 to access the sensor information as well as the data.

However, the use of the prototype does not just use a standardized interface without knowing the proprietary interface of the sensor. Using the prototype the system is more versatile. The different parts that compose the system can be replaced by other ones as long as they are compliant with the standard. For instance, one can change the accelerometer used in the study case for another accelerometer which is compliant with the standard.

The integration of the prototype with the game reduces the work of the implementation to the extent that knowledge of the manufacturer interface is not required; this feature is applicable to AAL solutions.

As described in the study case (Section 7) the sensor is added as a controller interface to the system as soon as it is turned on and within the network area coverage. This feature raises several interesting possibilities. Combining heterogeneous environmental sensors creates a more versatile solution [45] and this prototype does not just allow us to adding personal sensors, it also permits integration with environmental sensors. These sensors can be automatically incorporated into the system as soon as they are in the range area of the network (WSN).

## Figures and Tables

**Table 1.** TIMs autonomy. Initial and end time of the tests and the voltage of the battery.

**Initial Time**	**End Time**	**Initial Battery**	**End Battery**
1,367,908 s	1,367,987 s	3 V	1.5 V

**Table 2.** Results for the TOSSIM simulation with two variables: number of TIMs in the network and transmission frequency for each TIM. Results present the average messages received at the NCAP per each TIM.

**TIMs**	**30 ms**	**60 ms**	**90 ms**
1	2001	2001	2001
2	2000	2001	2001
3	1934	2001	2001
4	1603	2000	2001
5	1205	1968	2000
6	929	1897	2000
7	739	1801	1980
8	588	1573	1951
9	544	1307	1879
10	401	1090	1817
11	332	922	1749
12	275	775	1568
13	234	657	1382
14	199	567	1206
